# Glial autoantibody prevalence in Chinese optic neuritis with onset after age 45: clinical factors for diagnosis

**DOI:** 10.3389/fimmu.2023.1181908

**Published:** 2023-08-29

**Authors:** Honglu Song, Yucai Chuai, Mo Yang, Huanfen Zhou, Mingming Sun, Quangang Xu, Shihui Wei

**Affiliations:** ^1^ Department of Ophthalmology, The First Medical Center of the Chinese People's Liberation Army General Hospital, Beijing, China; ^2^ Department of Ophthalmology, Bethune International Peace Hospital, Shijiazhuang, Hebei, China; ^3^ Department of Special Medical Services, Bethune International Peace Hospital, Shijiazhuang, Hebei, China; ^4^ Department of Neuro-ophthalmology, Eye Hospital, China Academy of Chinese Medical Sciences, Beijing, China

**Keywords:** optic neuritis, middle-aged and elderly-onset, aquaporin 4, myelin oligodendrocyte glycoprotein, glial autoantibody, diagnostic test

## Abstract

**Purpose:**

As glial autoantibody testing is not yet available in some areas of the world, an alternative approach is to use clinical indicators to predict which subtypes of middle-aged and elderly-onset optic neuritis (ON) have manifested.

**Method:**

This study was a single-center hospital-based retrospective cohort study. Middle-aged and elderly-onset ON patients (age > 45 years) who had experienced the first episode of ON were included in this cohort. Single- and multi-parametric diagnostic factors for middle-aged and elderly-onset myelin oligodendrocyte glycoprotein immunoglobulin-associated ON (MOG-ON) and aquaporin-4 immunoglobulin-related ON (AQP4-ON) were calculated.

**Results:**

From January 2016 to January 2020, there were 81 patients with middle-aged and elderly-onset ON, including 32 (39.5%) AQP4-ON cases, 19 (23.5%) MOG-ON cases, and 30 (37.0%) Seronegative-ON cases. Bilateral involvement (47.4%, *P* = 0.025) was most common in the MOG-ON group. The presence of other concomitant autoimmune antibodies (65.6%, *P* = 0.014) and prior neurological history (37.5%, *P* = 0.001) were more common in the AQP4-ON group. The MOG-ON group had the best follow-up best-corrected visual acuity (BCVA) (89.5% ≤ 1.0 LogMAR, *P* = 0.001). The most sensitive diagnostic factors for middle-aged and elderly-onset MOG-ON were ‘follow-up VA ≤ 0.1 logMAR’ (sensitivity 0.89), ‘bilateral involvement or follow-up VA ≤ 0.1 logMAR’ (sensitivity 0.95), ‘bilateral involvement or without neurological history’ (sensitivity 1.00), and ‘follow-up VA ≤ 0.1 logMAR or without neurological history’ (sensitivity 1.00), and the most specific factor was ‘bilateral involvement’ (specificity 0.81). The most sensitive diagnostic factors for middle-aged and elderly-onset AQP4-ON were ‘unilateral involvement’ (sensitivity 0.88), ‘unilateral involvement or neurological history’ (sensitivity 0.91), and ‘unilateral involvement or other autoimmune antibodies’ (sensitivity 1.00), and the most specific factor was neurological history (specificity 0.98).

**Conclusion:**

Based on our cohort study of middle-aged and elderly-onset ON, MOG-ON is less prevalent than AQP4-ON and Seronegative-ON. Using multiple combined parameters improves the sensitivity and negative predictive value for diagnosing middle-aged and elderly-onset MOG-ON and AQP4-ON. These combined parameters can help physicians identify and treat middle-aged and elderly-onset ON early when glial autoantibody status is not available.

## Introduction

Optic neuritis (ON) is an inflammatory demyelinating lesion of the optic nerve and the leading cause of vision loss in young adults between 18 and 45 years old (1–3). However, there are limited studies describing the features of ON in populations older than 45 years of age. This study focuses on patients with ON who are over 45 years of age, and thus, the first onset of ON in this age group is referred to as middle-aged and elderly-onset ON. Recent advancements have established glial autoantibodies against aquaporin-4 immunoglobulin (AQP4-IgG) and myelin oligodendrocyte glycoprotein immunoglobulin (MOG-IgG) as biomarkers for atypical ON, significantly enhancing our understanding of ON (4, 5). The discovery that AQP4-IgG can differentiate neuromyelitis optica (NMO) from multiple sclerosis (MS) has led to the concept of neuromyelitis optica spectrum disorder (NMOSD) (6). The incidence of AQP4-ON and MOG-ON in the Caucasian population is significantly lower than that of MS-ON (7). Patients with MOG-IgG-positive ON (MOG-ON) tend to have a higher prevalence of bilateral optic nerve involvement, recurrence, optic disc edema, and favorable visual outcomes (8, 9). Differentiating between AQP4-ON and MOG-ON can be based on differences in clinical features, cerebrospinal fluid markers, disease severity and functional recovery, optical coherence tomography (OCT), and MRI manifestations (10–15).

While testing for MOG-IgG and AQP4-IgG has been recommended for identifying the classification of ON and guiding the treatment of different subtypes in recent years (6, 16), glial autoantibody detection is unavailable in certain areas, leading to delays in obtaining these results. Furthermore, there is limited research on middle-aged and elderly-onset ON (17, 18), and little is known about the clinical characteristics and prognosis of middle-aged and elderly-onset ON patients. A previous study included only 12 patients (10.9%) above the age of 50 in an investigation of acute ON, with only two cases being MOG-ON and one case being AQP4-ON (19). Given the differences in presentation between middle-aged and elderly-onset ON and early-onset ON (20, 21), our objective was to identify the clinical features of MOG-IgG and AQP4-IgG seropositive middle-aged and elderly-onset ON patients from a Chinese cohort (age > 45 years). Our study demonstrates that the subtypes of middle-aged and elderly-onset ON can be predicted, enabling precise diagnosis and early intervention even in the absence of glial autoantibody testing.

## Materials and methods

### Patient enrolment

We conducted a retrospective cohort study at tertiary centers between January 2016 and January 2020. The study included patients with middle-aged and elderly-onset optic neuritis (age of first onset > 45 years, with no upper age limit) who had experienced their initial acute ON attack in the Senior Department of Ophthalmology at the Chinese PLA General Hospital. This study was approved by the Ethics Committee of the Chinese PLA General Hospital (approval number S2019-111-01) and adhered to the principles of the Declaration of Helsinki and applicable Chinese laws. All selected patients received treatment with intravenous methylprednisolone (1000 mg/day) for three days, followed by a reduction in oral dose (initial dose of 1 mg/kg/day). The course of therapeutic intervention was adjusted based on the patient’s condition. Follow-up was conducted for all enrolled patients until the end of June 2021, with a minimum follow-up period of 3 months. Follow-up data were collected from clinical examinations during the patients’ follow-up period.

### Diagnostic criteria

The diagnosis of middle-aged and elderly-onset ON in all enrolled patients was confirmed by neuro-ophthalmologists. The inclusion criteria were as follows: (1) the middle-aged and elderly patients experienced the first episode of ON with acute loss of visual acuity or visual field in the presence or absence of eye pain, (2) the age of onset was >45 years old, (3) the patient presented with at least one visual abnormality of relative afferent pupillary defect, visual field defect, and abnormal visual evoked potential, and (4) regardless of whether they had a history of central nervous system (CNS) demyelination. The exclusion criteria were as follows: (1) presence of any other non-inflammatory optic neuropathy, such as ischemic, hereditary, metabolic, toxic, infectious, compressive, and infiltrative optic neuropathy; (2) presence of glaucoma, anterior segment diseases, and retinal or macular diseases; (3) unavailable glial autoantibody (MOG- and AQP4-IgG) status; and (4) inability to participate in a three-month follow-up.

### Neuro-ophthalmic and laboratory examinations

We recorded demographic and clinical manifestations, including gender, age at first onset of ON, previous neurological history, pain, optic disc swelling (ODS), laterality, concomitant presence of other autoimmune antibodies, best-corrected visual acuity (BCVA) at the nadir, immunosuppression therapy, follow-up visual acuity outcome, and recurrence of ON or CNS demyelinating manifestations elsewhere. The annualized relapse rate (ARR) was calculated by dividing the number of relapses by the total number of years since the first onset. The ARR was assessed only for patients who experienced a relapse during the study’s follow-up period. BCVA was assessed using the Snellen chart (15). BCVA values were transformed to the logarithm of the minimum angle of resolution (logMAR), where logMAR = -logBCVA. The following logMAR values were used to represent non-numeric visual acuity (22, 23): 3.00 logMAR for no light perception, 2.70 logMAR for light perception, 2.00 logMAR for hand movement, and 1.70 logMAR for counting fingers. Visual bad recovery was defined as severe vision loss that failed to reach a BCVA ≤0.1 (1.0 logMAR) at the follow-up (23). Follow-up VA referred to the BCVA outcome at least 3 months after the first optic neuritis attack and before the second ON attack.

Based on the status of serum glial autoantibodies, the enrolled patients were divided into three groups: MOG-ON, AQP4-ON, or double-antibody seronegative ON (Seronegative-ON). Serum glial autoantibodies were detected during each patient’s first acute ON episode. The titers of MOG-IgG and AQP4-IgG were measured using a commercial cell-based assay (CBA) or a domestic CBA (Euroimmun before September 2019 and Maiyuan Ltd afterward), as described in previous studies (24, 25). Serum samples were screened for other autoimmune antibodies, such as antinuclear antibodies (ANA), extractable nuclear antigen antibodies (SSA and SSB), anticardiolipin antibodies (ACLs and β2-GPI), anti-thyroid peroxidase autoantibody (anti-TPOAb), anti-thyroglobulin antibodies (ATAs), rheumatoid factor (RF), human leukocyte antigen B27 (HLA-B27), and anti-neutrophil cytoplasmic antibodies (ANCA) at the Examination Centre for Biomedical Research of the Chinese People’s Liberation Army General Hospital.

### Statistical analysis

The enrolled patients were divided into three subgroups based on the presence of glial autoantibodies. Demographic parameters were compared between the different subgroups. Quantitative variables were analyzed using Kruskal-Wallis H tests, and categorical variables were analyzed using chi-squared (*χ2*) tests or Fisher’s exact tests, as appropriate. Results with a two-tailed *P*-value less than 0.05 were considered statistically significant. Statistical analysis was conducted using IBM SPSS version 20.0 software.

## Results

### Demographics and clinical characteristics

**Figure 1:**
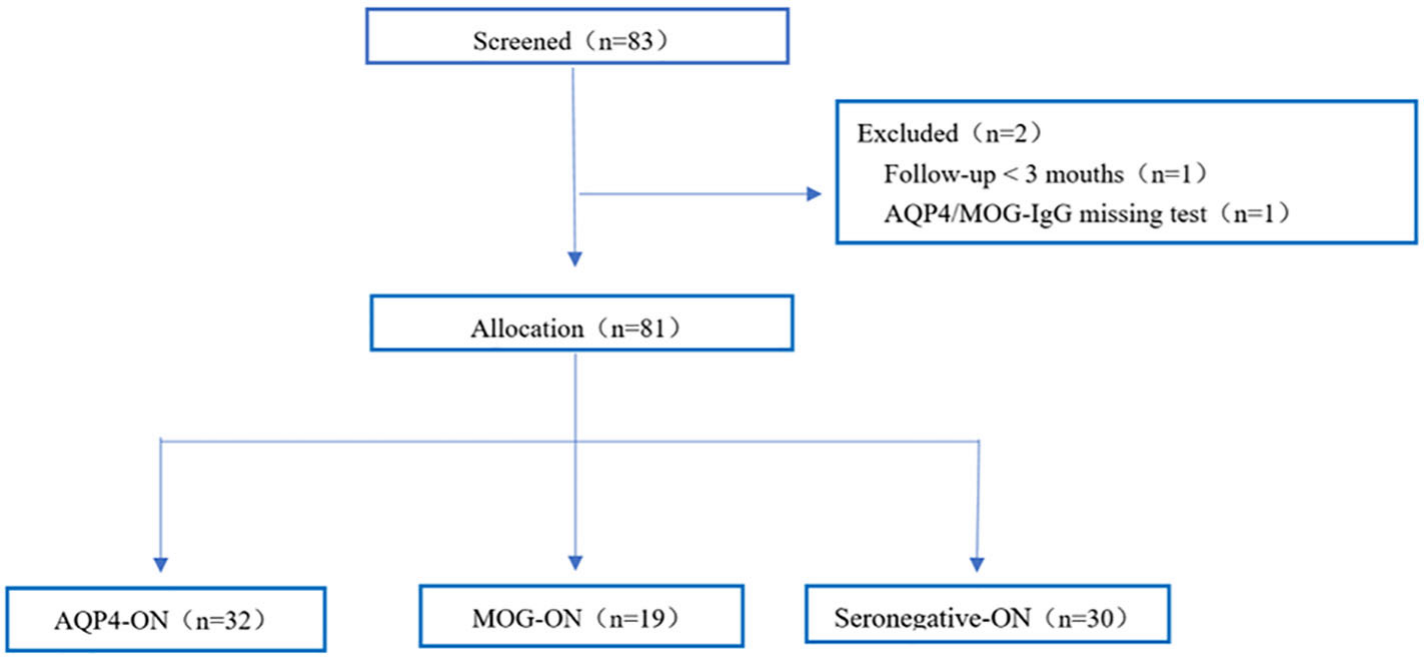
Study flowchart of 81 middle-aged and elderly-onset ON patients in this cohort.

Eighty-one middle-aged and elderly-onset ON patients were included in the final analysis (**Figure 1**). **Table 1** summarizes the clinical manifestations of the selected middle-aged and elderly-onset ON patients based on glial autoantibodies. Among the patients, 39.5% (32/81) were categorized as AQP4-ON, 23.5% (19/81) as MOG-ON, and 37.0% (30/81) as Seronegative-ON. The majority of the patients were women (87.7%), and there was no significant difference in the percentage of female patients among the three groups (AQP4-ON group: 87.5% women; MOG-ON group: 94.7% women; Seronegative-ON group: 83.3% women; *P=*0.570). The median age at ON onset did not significantly differ among the groups, with values of 51.0, 52.0, and 54.0 years in the MOG-ON, AQP4-ON, and Seronegative-ON subgroups, respectively (*P=*0.713). Optic disc swelling (ODS) was relatively more common in the MOG-ON group, but there was no significant difference in ODS among the 51 patients who recorded this symptom (*P=*0.649). The frequency of ocular pain during the first onset attack was similar among the three groups, as reported by the 71 patients who recorded this symptom (*P=*0.295). Bilateral optic nerve involvement was most common in the MOG-ON group (47.4%, *P*=0.025), followed by the Seronegative-ON group (26.7%) and the AQP4-ON group (12.5%). However, compared to the other two ON subgroups, the proportion of concomitant autoimmune antibodies in the AQP4-ON group was elevated (AQP4-ON 65.6% vs Seronegative-ON 40.0% vs MOG-ON 26.3%, *P=*0.014). Among the 13 patients with previous neurological history, 12 were AQP4-IgG seropositive (finally diagnosed with NMOSD) and were assigned to the AQP4-ON subgroup, while one Seronegative-ON patient, who was eventually diagnosed with multiple sclerosis, was assigned to the Seronegative-ON group. The most common prior neurological history was observed in the AQP4-ON group (37.5%, *P=*0.001), followed by the Seronegative-ON group (3.3%) and the MOG-ON group (0.0%). Only this one Seronegative-ON patient was ultimately diagnosed with MS-associated ON (MS-ON).

**Table 1:** Clinical characteristics of the enrolled middle-aged and elderly-onset ON patients based on glial autoantibodies.

	Total	AQP4-ON	MOG-ON	Seronegative-ON	*P* value
No. of patients	81	32	19	30	
Gender, women (%)	71 (87.7)	28 (87.5)	18 (94.7)	25 (83.3)	0.570*
Age at first ON, median (IQR)	53.5 (49.0, 57.0)	52.0 (49.0, 56.8)	51.0 (49.0, 57.0)	54.0 (50.5, 58.0)	0.713
Age at first ON, mean ± SD	54.3 ± 6.6	54.4 ± 7.4	53.5 ± 6.3	54.5 ± 6.0	0.845
Optic disc swelling (%)	32/51 (62.7)	9/16 (56.2)	8/11 (72.7)	15/24 (62.5)	0.649*
Pain (%)	50/71 (70.4)	19/28 (67.9)	16/19 (84.2)	15/24 (62.5)	0.295#
**Bilateral ON** (%)	21 (25.9)	4 (12.5)	9 (47.4)	8 (26.7)	***0.025*** *
**Other autoimmune antibodies** (%)	38 (46.9)	21 (65.6)	5 (26.3)	12 (40.0)	***0.014*** #
**Neurological history** (%)	13 (16.0)	12 (37.5)	0 (0.0)	1 (3.3)	0.001*
Worst VA at nadir, median (IQR)	2.0 (1.0,3.0)	2.0 (1.4,3.0)	1.75 (1.0,2.8)	2.8 (1.0,3.0)	0.533
Worst VA ≥1.7 LogMAR	51/74 (68.9)	22/29 (75.9)	12/18 (66.7)	17/27 (63.0)	0.563#
Follow-up VA, median (IQR)	1.2 (0.2,2.0)	1.5 (0.3,2.0)	0.2 (0.0,0.8)	1.5 (0.8,2.2)	***0.003***
**Follow-up VA≤1.0 LogMAR**	40 (49.4)	14 (43.8)	17 (89.5)	9 (30.0)	***0.001*** #
Immunosuppressant treatment (%)	26 (32.1)	17 (53.1)	3 (15.8)	6 (20.0)	***0.005*** #
Rituximab	9	6	2	1	
Mycophenolate mofetil	14	9	1	4	
Azathioprine	1	1	0	0	
Others	2	1	0	1	
Follow-up time, m, median (IQR)	12 (4.5,23.5)	11.5 (7.0,24.8)	7.0 (3.0,18.0)	15.5 (5.0,23.3)	0.438
Relapse (%)	36 (44.4)	14 (43.8)	9 (47.4)	13 (43.3)	0.999#
ARR, n, median (IQR)	0.8 (0.5,1.1)	0.7 (0.5,1.0)	1.1 (0.6,1.9)	0.8 (0.4,1.2)	0.371

*Fisher’s exact test. #χ^2^ test. Other P values are calculated using Kruskal-Wallis H tests. ARR, annual relapse rate; AQP4, Aquaporin-4; CNS, central nervous system; IQR, interquartile range; MOG, myelin oligodendrocyte glycoprotein; ON, optic neuritis; VA, visual acuity; P<0.05 is marked in bold and italic.

### Visual outcomes and clinical prognosis

The proportion of patients with the worst visual acuity (≥ 1.7 LogMAR) in middle-aged and elderly-onset ON was 68.9%. The severity of the worst visual acuity did not significantly differ among the three subgroups (*P*=0.533), but there was a significant difference in follow-up visual acuity (*P*=0.003). The MOG-ON group had the best follow-up visual acuity (89.5% ≤ 1.0 LogMAR), followed by the AQP4-ON group (43.8% ≤ 1.0 LogMAR), while the Seronegative-ON group had the worst follow-up visual acuity (30.0% ≤ 1.0 LogMAR, *P=*0.001). Among the patients, 17 out of 32 in the AQP4-ON group (53.1%), 3 out of 19 in the MOG-ON group (15.8%), and 6 out of 30 in the Seronegative-ON group (20.0%) received immunosuppressive therapy during the follow-up period. AQP4-ON patients had the highest proportion of receiving immunosuppressive therapy compared to the other two subgroups (*P=*0.005), with rituximab and mycophenolate mofetil being the two most common treatment options. During a median follow-up of 12 months, 43.8% of AQP4-ON patients experienced ON recurrence or CNS demyelinating events elsewhere, with an annualized relapse rate (ARR) of 0.7, compared to the Seronegative-ON group (ARR 0.8, recurrence rate 43.3%) and the MOG-ON group (ARR 1.1, recurrence rate 47.4%).

### Predicting middle-aged and elderly-onset MOG-ON

In predicting middle-aged and elderly-onset MOG-ON, the most sensitive clinical features were sex (women: NPV 0.90, sensitivity 0.95), follow-up VA ≤0.1 logMAR (NPV 0.95, sensitivity 0.89), pain (NPV 0.86, sensitivity 0.84), and ODS (NPV 0.84, sensitivity 0.73). The most specific values were bilateral optic nerve involvement and prior neurological history, with specificities of 0.81 and 0.79, respectively (**Table 2**). Using the parallel composite calculation of ‘ODS or bilateral’, ‘ODS or bilateral or relapse’, ‘bilateral or follow-up VA ≤0.1 logMAR’, ‘bilateral or without neurological history’ or ‘follow-up VA ≤0.1 logMAR or without neurological history’, the sensitivity increased to 0.91, 0.91, 0.95, 1.00, and 1.00, respectively, and the negative predictive value (NPV) increased to 0.92, 0.91, 0.97, 1.00, and 1.00, respectively, as shown in the last five rows of **Table 2**.

**Table 2:** Performance of clinical features for predicting MOG-ON among all middle-aged and elderly-onset ON patients*.

	Sensitivity (95% CI)	Specificity (95% CI)	PPV (95% CI)	NPV (95% CI)
Female	0.95 (0.72-1.00)	0.15 (0.07-0.26)	0.25 (0.16-0.37)	0.90 (0.54-0.99)
**Bilateral ON**	0.47 (0.25-0.71)	**0.81 (0.68-0.89)**	0.43 (0.22-0.66)	0.83 (0.71-0.91)
Pain	0.84 (0.60-0.96)	0.35 (0.22-0.49)	0.32 (0.20-0.47)	0.86 (0.63-0.96)
ODS	0.73 (0.39-0.93)	0.40 (0.25-0.57)	0.25 (0.12-0.44)	0.84 (0.60-0.96)
Neurological history	0.00 (0.00-0.21)	0.79 (0.66-0.88)	0.00 (0.00-0.28)	0.72 (0.60-0.82)
Other autoimmune antibodies	0.26 (0.10-0.51)	0.47 (0.34-0.60)	0.13 (0.05-0.29)	0.67 (0.51-0.80)
Worst VA ≥1.70 logMAR	0.67 (0.41-0.86)	0.30 (0.19-0.44)	0.24 (0.13-0.38)	0.74 (0.51-0.89)
**Follow-up VA ≤1.0 logMAR**	**0.89 (0.65-0.98)**	0.63 (0.50-0.75)	0.43 (0.27-0.59)	**0.95 (0.82-0.99)**
Relapse	0.47 (0.25-0.71)	0.56 (0.43-0.69)	0.25 (0.13-0.43)	0.78 (0.63-0.88)
ODS or bilateral	0.91 (0.57-1.00)	0.28 (0.15-0.44)	0.26 (0.14-0.42)	0.92 (0.60-1.00)
ODS or bilateral or relapse	0.91 (0.57-1.00)	0.25 (0.13-0.42)	0.25 (0.13-0.42)	0.91 (0.57-1.00)
**Bilateral or follow-up VA≤0.1 logMAR**	**0.95 (0.72-1.00)**	0.51 (0.37-0.63)	0.37 (0.24-0.52)	**0.97 (0.82-1.00)**
**Bilateral or without neurological history**	**1.00 (0.79-1.00)**	0.18 (0.10-0.30)	0.27 (0.18-0.40)	**1.00 (0.68-1.00)**
**Follow-up VA ≤0.1 logMAR or without neurological history**	**1.00 (0.79-1.00)**	0.11 (0.05-0.22)	0.26 (0.17-0.37)	**1.00 (0.56-1.00)**

*Disease-absent group included the AQP4-ON and Seronegative-ON patients. AQP4, Aquaporin-4; CI, confidence interval; MOG, myelin oligodendrocyte glycoprotein; NPV, negative predictive value; ON, optic neuritis; ODS, optic disc swelling; PPV, positive predictive value; VA, visual acuity.

Data in bold indicate high sensitivity and specificity.

### Predicting middle-aged and elderly-onset AQP4-ON

In order to predict middle-aged and elderly-onset AQP4-ON (**Table 3**), the most sensitive predictors were unilateral involvement (NPV 0.81, sensitivity 0.88), sex (women: NPV 0.60, sensitivity 0.88), and other concomitant autoimmune antibodies (NPV 0.74, sensitivity 0.66), while a neurological history (PPV 0.92, specificity 0.98) was the most specific predictor. Using the parallel test ‘unilateral or neurological history’ improved sensitivity to 0.91 with the optimal NPV of 0.84. Parallel testing using ‘unilateral or other autoimmune antibodies’ increased the sensitivity to 1.00, with an optimal NPV of 1.00.

**Table 3:** Performance of clinical features for predicting AQP4-ON among all middle-aged and elderly-onset ON patients*.

	Sensitivity (95% CI)	Specificity (95% CI)	PPV (95% CI)	NPV (95% CI)
Female	0.88 (0.70-0.96)	0.12 (0.05-0.25)	0.39 (0.28-0.52)	0.60 (0.27-0.86)
**Unilateral ON**	**0.88 (0.70-0.96)**	0.35 (0.22-0.50)	0.47 (0.34-0.60)	**0.81 (0.57-0.94)**
Pain	0.68 (0.48-0.83)	0.28 (0.16-0.44)	0.38 (0.25-0.53)	0.57 (0.34-0.77)
ODS	0.56 (0.31-0.79)	0.34 (0.20-0.52)	0.28 (0.14-0.47)	0.63 (0.39-0.83)
Neurological history	0.38 (0.22-0.56)	**0.98 (0.88-1.00)**	**0.92 (0.62-1.00)**	0.71 (0.58-0.81)
Other autoimmune antibodies	0.66 (0.47-0.81)	0.65 (0.50-0.78)	0.55 (0.38-0.71)	0.74 (0.58-0.86)
Worst VA ≥1.70 logMAR	0.76 (0.56-0.89)	0.36 (0.23-0.52)	0.44 (0.30-0.59)	0.70 (0.47-0.86)
Follow-up VA >1.0 logMAR	0.56 (0.38-0.73)	0.53 (0.38-0.67)	0.44 (0.29-0.60)	0.65 (0.48-0.79)
Relapse	0.44 (0.27-0.62)	0.55 (0.40-0.69)	0.39 (0.24-0.56)	0.60 (0.44-0.74)
**Unilateral or neurological history**	**0.91 (0.74-0.98)**	0.33 (0.20-0.48)	0.47 (0.34-0.60)	**0.84 (0.60-0.96)**
**Unilateral or other autoimmune antibodies**	**1.00 (0.87-1.00)**	0.22 (0.12-0.37)	0.46 (0.34-0.58)	**1.00 (0.68-1.00)**

*Disease-absent group included the MOG-ON and Seronegative-ON patients. AQP4, Aquaporin-4; CI, confidence interval; MOG, myelin oligodendrocyte glycoprotein; NPV, negative predictive value; ON, optic neuritis; ODS, optic disc swelling; PPV, positive predictive value; VA, visual acuity.

Data in bold indicate high sensitivity and specificity.

## Discussion

We previously discovered that utilizing multiple parameters can enhance the specificity and sensitivity in diagnosing AQP4-ON and MOG-ON in Chinese children, aiding ophthalmologists in diagnosing and treating pediatric ON when the status of glial autoantibodies is unknown (24). In another previous study on the prevalence of glial autoantibody-mediated ON and clinical predictive factors for diagnosing MOG-ON in adult patients, the combined clinical factor ‘ODS or bilateral or recurrent ON’ exhibited the highest sensitivity and an NPV of 100% (26). However, middle-aged and elderly-onset ON differs in clinical features and prognosis when compared to early-onset ON (20, 21). In this study, we compared differences in ON clinical features based on serum glial autoantibody status to identify specific single or combined clinical indicators for diagnosing ON subtypes. This approach is expected to provide accurate diagnosis and early intervention without the need for glial autoantibody detection.

In our middle-aged and elderly-onset ON cohort, 39.5% of the participants had AQP4-ON, 23.5% had MOG-ON, and 37.0% had Seronegative-ON. Among them, only one Seronegative-ON patient was eventually diagnosed with MS-ON. MS-ON is the most common subtype of demyelinating ON in Caucasians but is relatively rare among Asians (19). Conversely, MOG-ON is quite prevalent in middle-aged and elderly-onset ON patients (23.5%) compared to MS-ON (1.2%). These findings confirm previous estimates of MOG-ON prevalence among ON, ranging from 10% to 30.8% (19, 27–29). However, the prevalence of AQP4-ON in the Chinese population (35.0-42.4%) (27, 28) is higher than in Europeans (4.5%) (19). The proportion of AQP4-ON observed in this study (39.5%) aligns with these figures.

Vision loss caused by MOG-ON and AQP4-ON can be severe, but the recovery of patients with MOG-ON is typically better than that of patients with AQP4-ON (8, 23, 30). In this study, patients with MOG-ON exhibited the best final BCVA (89.5%≤1.0 LogMAR), followed by patients with AQP4-ON (43.8%≤1.0 LogMAR), while patients with Seronegative-ON had the worst final BCVA (30.0%≤1.0 LogMAR). These results are consistent with previous findings that approximately 6-13% of MOG-ON patients have a final BCVA below 20/200, whereas the proportion of AQP4-ON patients is close to half (8, 23, 30, 31).

MOG-IgG testing is recommended for cases of ODS, bilateral optic nerve involvement, recurrent attacks, severe visual impairment, and steroid-dependent relapse in acute ON patients (16). Based on retrospective studies, it is impossible to predict the prognosis of ON patients based solely on these clinical features or infer which antibody is positive based on clinical features. Performing MOG-Ab testing only in adult patients with ODS, bilateral ON, or recurrent ON would limit the risk of missing any MOG-ON cases to less than half (45%) of all patients without missing any MOG-ON cases (26). In this study, we found that the combination of ODS with bilateral involvement and/or relapse of ON was highly sensitive for predicting middle-aged and elderly-onset MOG-ON. Consistent with previous literature (25, 26), bilateral optic nerve involvement and better visual prognoses in patients with MOG-ON were more common compared to those with AQP4-ON or Seronegative-ON, supporting our findings that combined bilateral optic nerve involvement or follow-up VA≤0.1 logMAR had high sensitivity and NPV in diagnosing MOG-ON. Our study also showed that patients with middle-aged and elderly-onset AQP4-ON were more likely to have a history of neurological involvement than patients with MOG-ON or Seronegative-ON. Therefore, patients without a neurological history coupled with bilateral involvement or with follow-up VA ≤0.1 logMAR had higher sensitivity and NPV in the diagnosis of MOG-ON. We recommend performing MOG-IgG detection in middle-aged and elderly-onset ON patients with two clinical indicators: ‘bilateral or follow-up VA ≤0.1 logMAR or without neurological history’ to achieve a missed diagnosis rate of less than 5%.

NMOSD is often associated with autoimmune diseases such as systemic lupus erythematosus, Sjögren’s syndrome, and Hashimoto’s disease (32). Nearly half of AQP4-ON patients exhibit additional autoimmune antibodies, including serum ANA, anti-SSA antibodies, anti-SSB antibodies, anti-TPOAb, and ATAs (29). We found that the presence of additional autoimmune antibodies was most common in the AQP4-ON group (65.6%). Although approximately 70% of patients with AQP4-NMOSD experience CNS attacks during the onset episodes, the majority of these attacks are isolated CNS attacks (33) and are not limited to the optic nerve. Prior neurological history was most common in patients with AQP4-ON (37.5%). To predict middle-aged and elderly-onset AQP4-ON, sex, unilateral involvement, and other autoimmune antibodies were the more sensitive predictors, while neurological history was the most specific predictor. Evaluating whether the patients had unilateral involvement or prior neurological history improved sensitivity. Parallel testing using unilateral involvement or other autoimmune antibodies further increased sensitivity. Detecting AQP4-IgG in middle-aged and elderly-onset ON patients with ‘unilateral or neurological history’ and ‘unilateral or other autoimmune antibodies’ resulted in missed diagnosis rates of 9% and 0%, respectively. Therefore, we recommend that middle-aged and elderly-onset ON patients with a neurological history should be treated according to AQP4-ON if the AQP4-IgG status is unknown.

The diagnostic indicators selected in this article have certain limitations. Firstly, this study was conducted at a single center, which may introduce patient selection bias and limit the generalizability of the findings to the broader population. Our center, being a larger neuro-ophthalmic center in China, may have a patient population with more severe middle-aged and elderly-onset ON conditions. Secondly, the presence of incomplete medical records documenting ocular pain and ODS in some patients may have introduced bias into our research results. In previous literature, ODS has been found to account for a high proportion of MOG-ON cases, but this result was not observed in our middle-aged and elderly-onset ON patients. This discrepancy may be related to the incomplete recording of ODS symptoms in some patients’ medical records. Thirdly, we did not specifically investigate the differences in MRI and OCT abnormalities among patients in the middle-aged and elderly-onset ON subgroups, mainly due to the lack of complete MRI and OCT data for many patients. Therefore, further prospective large-sample studies are needed to evaluate the diagnostic predictive value of MRI and OCT in middle-aged and elderly-onset ON. Fourth, it is generally believed that the positive rate of GFAP antibody in cerebrospinal fluid is higher than in blood (34). However, as an ophthalmology center, obtaining cerebrospinal fluid samples is challenging. Although we cannot exclude the possibility of individual GFAP-positive ON patients, the lack of detection of this antibody is expected to have a negligible impact on the results of this study. Finally, our conclusions need to be further evaluated in future prospective multicenter clinical studies, and it would be beneficial to include a larger sample size of patients with middle-aged and elderly-onset ON from diverse ethnic and racial backgrounds in future research.

## Conclusion

Our study on the middle-aged and elderly-onset ON cohort yielded several key findings: (1) The prevalence of MOG-ON is lower compared to AQP4-ON and Seronegative-ON (23.5% vs. 39.5% vs. 37.0%), (2) MOG-ON patients demonstrate the best follow-up visual acuity, and (3) utilizing combinations of parameters enhances the sensitivity and negative predictive value in diagnosing middle-aged and elderly-onset MOG-ON or AQP4-ON. These initial findings are valuable in aiding physicians in distinguishing and diagnosing ON subtypes, as well as guiding treatment decisions for middle-aged and elderly-onset ON cases when glial autoantibody status is unavailable.

## Data availability statement

The original contributions presented in the study are included in the article/supplementary material. Further inquiries can be directed to the corresponding author.

## Ethics statement

The studies involving humans were approved by the Ethics Committee of the Chinese PLA General Hospital (approval number S2019-111-01). The studies were conducted in accordance with the local legislation and institutional requirements. The participants provided their written informed consent to participate in this study.

## Author contributions

This study was designed and implemented by HS, YC, and SW. Data collection, analysis, management, and interpretation of the data were performed by HS, YC, MY, HZ, MS, and QX. The manuscript was prepared by HS and YC. SW made critical revisions to the manuscript. All authors contributed to the article and approved the submitted version.
